# Progressive brachial plexus enlargement in hereditary transthyretin amyloidosis

**DOI:** 10.1007/s00415-021-10754-9

**Published:** 2021-08-19

**Authors:** Alessandro Salvalaggio, Daniele Coraci, Laura Obici, Mario Cacciavillani, Marco Luigetti, Anna Mazzeo, Francesca Pastorelli, Marina Grandis, Tiziana Cavallaro, Giulia Bisogni, Alessandro Lozza, Chiara Gemelli, Luca Gentile, Massimo Russo, Mario Ermani, Gian Maria Fabrizi, Rosaria Plasmati, Federica De Napoli, Marta Campagnolo, Francesca Castellani, Fabrizio Salvi, Silvia Fenu, Grazia Devigili, Davide Pareyson, Roberto Gasparotti, Claudio Rapezzi, Carlo Martinoli, Luca Padua, Chiara Briani

**Affiliations:** 1 Department of Neurosciences, University of Padova, Via Giustiniani 5, 35128 Padova, Italy; 2 Padova Neuroscience Center (PNC), University of Padova, Padova, Italy; 3 Neuroriabilitazione Ad Alta Intensità, Fondazione Policlinico Universitario A. Gemelli IRCCS, Rome, Italy; 4 Amyloidosis Research and Treatment Centre, Fondazione IRCCS Policlinico San Matteo, Pavia, Italy; 5 CEMES-EMG Lab, Synlab Group, Padova, Italy; 6 Neurology Unit, Fondazione Policlinico Universitario Gemelli IRCCS, Rome, Italy; 7 Department of Geriatrics, Neurosciences and Orthopaedics, Catholic University of the Sacred Heart, Rome, Italy; 8 Unit of Neurology and Neuromuscular Diseases, Department of Clinical and Experimental Medicine, University of Messina, Messina, Italy; 9 Division of Neurology, IRCCS Institute of Neurological Sciences, Bellaria Hospital, Bologna, Italy; 10 Department of Neuroscience, Rehabilitation, Ophthalmology, Genetics, Maternal and Child Health (DiNOGMI), University of Genova, Genova, Italy; 11 Ospedale Policlinico San Martino IRCCS, Genova, Italy; 12 Neurology Unit, Department of Neuroscience, Biomedicine and Movement Sciences, University of Verona, Verona, Italy; 13 Centro Clinico NEMO Adulti, Rome, Italy; 14 Rare Neurodegenerative and Neurometabolic Diseases Unit, Department of Clinical Neurosciences, Fondazione IRCCS Istituto Neurologico Carlo Besta, Milan, Italy; 15 Neurological Unit I, Department of Clinical Neurosciences, Fondazione IRCCS Istituto Neurologico Carlo Besta, Milan, Italy; 16 Department of Medical and Surgical Specialties, Radiological Sciences, and Public Health, University of Brescia, Brescia, Italy; 17 Cardiological Center, University of Ferrara, Ferrara, Italy; 18 Maria Cecilia Hospital, GVM Care & Research, Cotignola, RA Italy; 19 Department of Scienze Della Salute, University of Genova, Genova, Italy

**Keywords:** Amyloidosis, Transthyretin, Brachial plexus, Ultrasound, Peripheral nerves

## Abstract

Axonal polyneuropathy is the main feature of hereditary transthyretin amyloidosis (ATTRv). Nerve morphological abnormalities have been reported, but longitudinal changes have never been assessed. We performed a prospective widespread nerve ultrasound evaluation and nerve cross-sectional area (CSA) was compared with baseline data in both ATTRv patients and pre-symptomatic carriers. Thirty-eight subjects were evaluated (mean follow-up 17.1 months), among them 21 had polyneuropathy while 17 were pre-symptomatic carriers. CSA significantly increased at brachial plexus in both groups (*p* = 0.008 and *p* = 0.012) pointing to progressive brachial plexus enlargement as a longitudinal biomarker of both disease progression and disease occurrence in pre-symptomatic carriers.

## Introduction

The most common manifestation of hereditary transthyretin amyloidosis (ATTRv, v for variant) in non-endemic regions is a length-dependent axonal sensorimotor polyneuropathy (PN) [1]. Lately, the demand of disease biomarkers is emerging to (i) monitor the disease course and (ii) to identify early pathological signs in pre-symptomatic carriers [2]. The former issue is required also for monitoring the response to treatment, while the latter is crucial for early diagnosis and timely therapy [3–5]. Previous findings suggest that subtle nerve abnormalities may precede the clinical or neurophysiological demonstration of polyneuropathy [6–8], but the time-course of the early abnormalities in pre-symptomatic carriers has never been addressed. Recently, we have demonstrated at nerve ultrasound (US), an enlargement of brachial plexus in ATTRv-PN patients, but no in pre-symptomatic carriers, pointing to brachial plexus enlargement as a possible morphological biomarker of the disease [9].

We now report on nerve US follow-up study in the same cohort of patients and in pre-symptomatic carrier to evaluate whether morphological changes may mirror disease progression.

## Methods

**Fig. 1:**
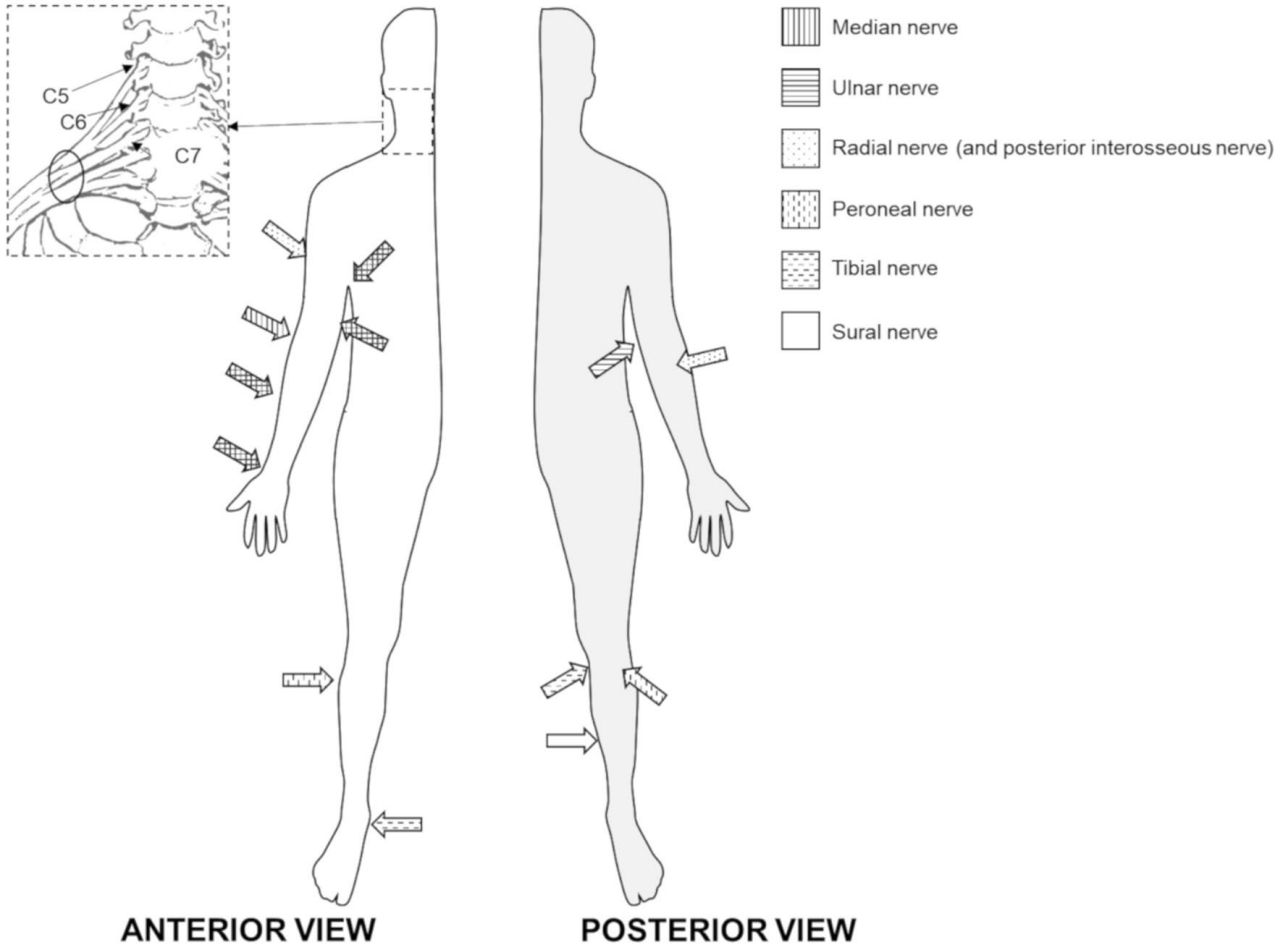
Schematic representation of the site of ultrasound evaluation along the course of nerves

**Fig. 2:**
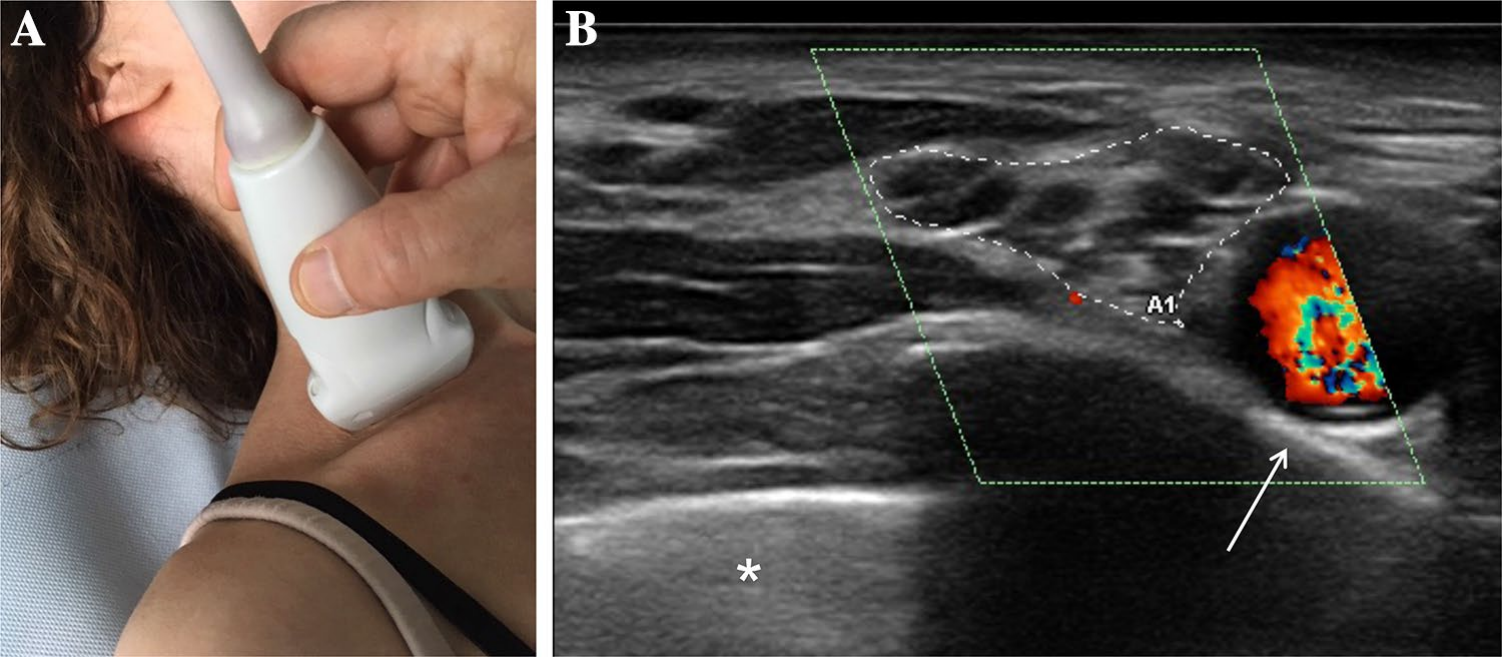
**a** Position of the probe in the US evaluation of right brachial plexus. **b** Right brachial plexus (contoured by dot line) in a healthy control, subclavian artery is visualized in color mode. Arrow points out the first rib, * is positioned on the lung

**Fig. 3:**
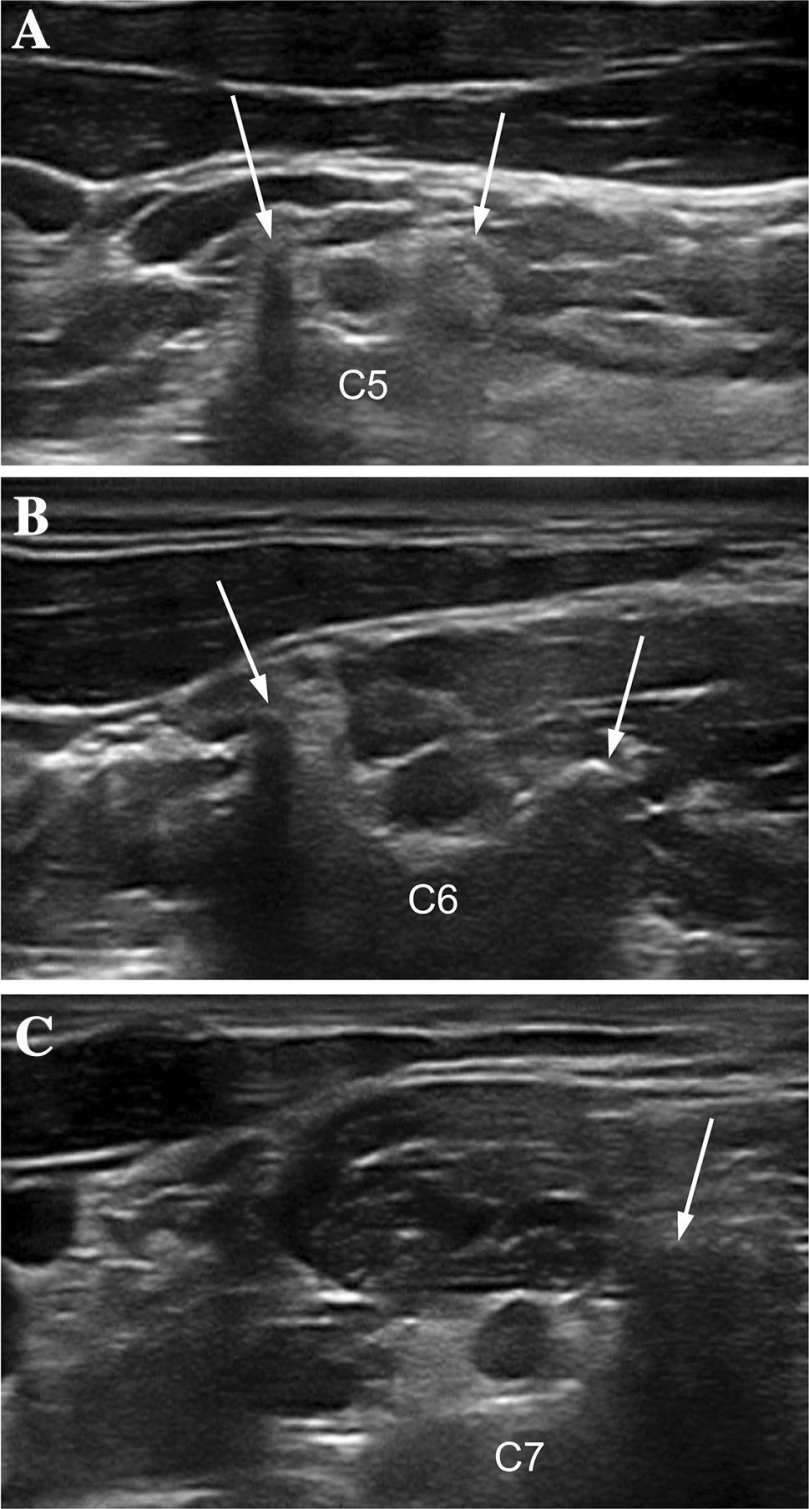
C5, C6 and C7 US appearance in a healthy control. Nerve roots are contoured by a circle, arrows point out the transvers processes (posterior tubercle)

Both ATTRv-PN patients and pre-symptomatic carriers with mutated TTR gene aged > 18 years were recruited from seven Italian centers. Subject with diabetes mellitus or other conditions possible cause of neuropathy were excluded. The baseline cohort (62 subjects) and the clinical, neurophysiological and US evaluations have previously been described [9]. US evaluation was performed by two neurophysiologists (DC, MC) with expertise in nerve ultrasound with a US system equipped with high-frequency linear transducer, frequency range 10–18 MHz (MyLab Seven Esaote, Genova, Italy and Toshiba Aplio 400). The probe was kept perpendicular to the nerves. The best visualized cross-sectional area (CSA) was measured with the ‘‘ellipse method’’ when applicable or the ‘‘tracing method’’ when the nerve had an irregular shape. The mean CSA value of three measurements was considered. Follow-up US evaluation was performed for each subject by the same neurophysiologist who performed the baseline evaluation. The course of median and ulnar nerves was followed bilaterally from axilla to wrist; measurement of nerve CSA at wrist, forearm, and arm were performed. Ulnar nerve CSA was measured also at the elbow (see below). The course of peroneal nerve was followed bilaterally from the popliteal fossa to the proximal third of the leg with measurement of the nerve CSA at popliteal fossa. Brachial plexus was measured at supraclavicular space at the level of divisions, after the trunks and before the cords. The course of tibial nerve was followed bilaterally in the popliteal fossa with measurement of the nerve CSA. The course of sural nerve was followed bilaterally from the median third of the leg to the malleolus with measurement of the nerve CSA at the median third of the leg. The following nerve trunks were evaluated bilaterally (Fig. 1): median nerve at wrist, forearm, elbow, arm and axilla; ulnar nerve at wrist, forearm, elbow, arm and axilla; posterior interosseous nerve at forearm; radial nerve at spiral groove; fibular nerve at fibular head and popliteal fossa; tibial nerve at the ankle and popliteal fossa; sciatic nerve at proximal thigh; sural nerve at the distal calf; brachial plexus at supraclavicular space (Fig. 2); C5, C6 and C7 roots after leaving transversal processes (Fig. 3).

Statistical analyses. Normality was tested with Kolmogorov–Smirnov method and variance equality with Levine test. Group comparison were performed with Mann–Whitney test for ordinal variables and two-tails student T test for normal distributed variables in independent groups or paired groups. Wilcoxon signed rank test was performed when the distribution of the difference between two samples’ means cannot be assumed to be normally distributed. Linear correlation between two variables was assessed with Pearson r when both the variables presented with a normal distribution. Significant level was set at *p* < 0.05. Bonferroni correction was applied for multiple comparisons. IBM SPSS Statistic version 23 was used for statistical analyses.

## Results

### Subjects

Thirty-eight subjects (22 men; mean age 59.2 years; mean body max index, BMI 26.0) were evaluated at follow-up (demographic data are reported in Table 1). The most represented TTR mutations were Phe64Leu (13 subjects), Val30Met (7 subjects), Glu89Gln (6 subjects) and Ile68Leu (4 subjects). 17 subjects (45%) were pre-symptomatic carriers (7 men; mean age 53.5; mean BMI 27.0), and according to the neurophysiologic findings, 21 (55%) ATTRv subjects had axonal polyneuropathy (15 men; mean age 63.9; mean BMI 25.2, 17 FAP stage 1, 4 FAP stage 2). In ATTRv patients, mean Neuropathy Impairment Score at lower limbs (NIS-LL) change was 4.7 ± 9.8, ranging from − 3 to 37. As expected, pre-symptomatic carriers were younger than symptomatic patients (*p* = 0.02), while BMI did not differ in the two groups (*p* = 0.28).

**Table 1:** Demographic data of the study cohort

	All cohort	ATTRv-PN	Carriers
Number	38	21	17
Sex	22 men, 16 women	15 men, 6 women	7 men, 10 women
Age	59.2 years (± 13.2) range 36–78	63.9 years (± 11.4) range 36–78	53.5 years (± 13.4) range 37–77
BMI	26.0 (± 5.2) range 18.8–38.1	25.2 range 18.8–38.1	27.0 range 19.5–35.2
TTR mutations	Phe64Leu (13 subjects), Val30Met (7), Glu89Gln (6), Ile68Leu (4), Thr49Ala (2), Tyr98Phe (2), Glu62Lys (1), Ala120Ser (1), Arg47Thr (1), Gly47Ala (1)	Val30Met (5), Glu89Gln (5), Phe64Leu (4 subjects), Ile68Leu (2), Thr49Ala (2), Tyr98Phe (2), Ala120Ser (1)	Phe64Leu (9 subjects), Val30Met (2), Ile68Leu (2), Glu89Gln (1), Glu62Lys (1), Arg47Thr (1), Gly47Ala (1)
Follow-up	17.1 months (± 3.8) range 6–28	17.0 months (± 4.4) range 6–28	17.3 months (± 3.0) range 10–20
NIS-LL baseline		20.0 (± 14.3)	
NIS-LL change		4.7 (± 9.8) range − 3/37	

Mean follow-up was 17.1 months (± 3.8), 17.0 months (± 4.4) in ATTRv patients, and 17.3 months (± 3.0) in pre-symptomatic carriers with no differences in the two groups (*p* = 0.23).

**Fig. 4:**
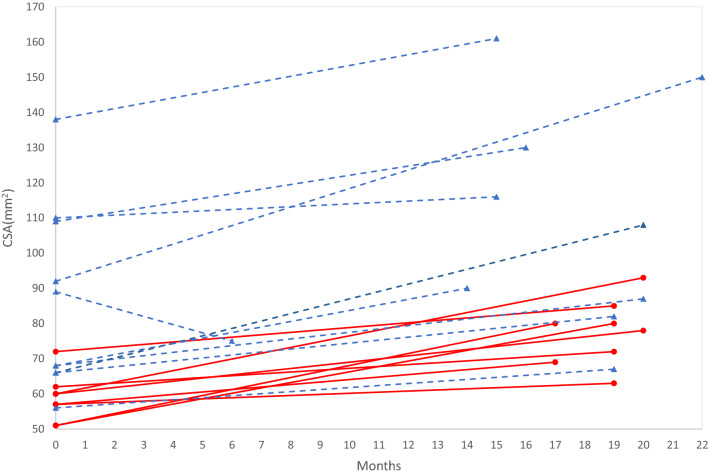
Brachial plexus CSA values at baseline and follow-up. Dotted lines represent ATTRv-PN patients and solid lines represent pre-symptomatic carriers. *CSA* cross-sectional area

Differences in the nerve CSA values between the follow-up and the baseline evaluations are reported in Tables 2 (all subjects) and 3 (ATTR-PN and carriers). Delta CSA (nerve CSA at follow-up – nerve CSA at baseline), when positive, represents an increase in nerve CSA. The average value between right and left side was considered for each site of each subjects. The brachial plexus CSA (identified as a possible biomarker for ATTRv-PN) [9], at follow-up, significantly increased (23.8%) when considering the whole cohort (*p* < 0.0001), but also the ATTR-PN patients (*p* = 0.008) and the pre-symptomatic carriers independently (*p* = 0.012). Changes in nerve CSA between the two groups scattered differed (i.e., median nerve at wrist and axilla, ulnar nerve at arm, C7 root) but these differences did not survive at multiple comparison correction. Notably, increase CSA at brachial plexus did not differ between ATTR-PN patients and pre-symptomatic carriers. CSA values of brachial plexus at baseline and follow-up are reported in Fig. 4.

**Table 2:** All subjects CSA data. Delta CSA (CSA at follow-up – CSA at baseline) at nerve ultrasound in ATTRv patients with polyneuropathy (PN) and pre-symptomatic carriers

Site	*N*	Mean delta CSA (mm^2^)	SD (mm^2^)	Mean delta CSA/mean baseline CSA × 100 (%)	
Median nerve at wrist	38	0.16	1.85	1.7	
Median nerve at forearm	38	− 0.13	1.53	− 2.1	
Median nerve at elbow	38	0.11	1.92	1.2	
Median nerve at arm	38	− 0.30	1.55	− 3.2	
Median nerve at axilla	34	− 0.69	2.15	− 7.1	
Ulnar nerve at wrist	38	− 0.38	1.16	− 7.5	
Ulnar nerve at forearm	38	− 0.24	1.01	− 4.2	
Ulnar nerve at elbow	38	0.29	2.06	3.3	
Ulnar nerve at arm	38	0.05	1.60	0.7	
Ulnar nerve at axilla	34	− 0.16	1.35	− 2.4	
Radial nerve at spiral groove	38	0.18	1.42	3.5	
Posterior interosseous nerve	37	− 0.05	0.963	− 2.2	
Brachial plexus at supraclavicular space*	**18**	**19,64**	**6.98**	**23.8**	***p* < 0.0001**
C5 root	33	0.28	1.51	4.4	
C6 root	33	− 0.12	2.30	− 1.4	
C7 root	31	− 0.40	1.68	− 4.1	
Fibular nerve at fibular head	38	− 0.21	1.77	− 2.4	
Fibular nerve at popliteal fossa	38	− 0.55	1.27	− 7.6	
Tibial nerve at tarsal tunnel	34	1.37	2.79	13.9	
Tibial nerve at popliteal fossa	35	− 0.10	6.11	− 0.4	
Sural nerve	38	0.02	0.56	1.1	
Sciatic nerve at mid-thigh	18	2.52	6.98	6.4	

The average values between right and left side are reported. Positive values mean an increase of CSA, negative values a decrease

*SD* standard deviation, *PN* polyneuropathy, *N* number of subjects with a CSA measurement; *p < 0.0001

**Table 3:** Delta CSA data of the two groups (ATTRv-PN and carriers). *P* values did not survive after correction for multiple comparisons

Site	Mean (SD) (mm^2^)		
	ATTRv-PN (*N* = 21)	ATTRv carriers (*N* = 17)	
Median nerve at wrist	*n* = − 0.56 (1.86) *n* = 21	1.04 (1.45) *n* = 17	*p* = 0.006
Median nerve at forearm	− 0.50 (1.55) *n* = 21	0.32 (1.42) *n* = 17	*p* = 0.100
Median nerve at elbow	− 0.18 (2.09) *n = *21	0.48 (1.66) *n = *17	*p* = 0.295
Median nerve at arm	− 0.46 (1.63) *n = *21	− 0.10 (1.47) *n = *17	*p* = 0.483
Median nerve at axilla	− 1.62 (2.44) *n = *17	0.24 (1.32) *n = *17	*p* = 0.009
Ulnar nerve at wrist	− 0.56 (0.95) *n = *21	− 0.15 (1.37) *n = *17	*p* = 0.285
Ulnar nerve at forearm	− 0.45 (1.12) *n = *21	0.02 (1.00) *n = *17	*p* = 0.184
Ulnar nerve at elbow	− 0.02 (1.59) *n = *21	0.67 (2.52) *n = *17	*p* = 0.314
Ulnar nerve at arm	− 0.56 (1.12) *n = *21	0.80 (1.79) *n = *17	*p* = 0.007
Ulnar nerve at axilla	− 0.56 (1.10) *n = *17	0.24 (1.48) *n = *17	*p* = 0.083
Radial nerve at spiral groove	0.11 (1.39) *n = *21	0.27 (1.49) *n = *17	*p* = 0.740
Posterior interosseous nerve	− 0.13 (1.19) *n = *20	0.05 (0.63) *n = *17	*p* = 0.591
Brachial plexus at supraclavicular space	20.40 (19.22) *n = *10	18.69 (10.14) *n = *8	*p* = 0.823
C5 root	0.25 (1.27) *n = *17	0.31 (1.77) *n = *16	*p* = 0.908
C6 root	− 0.04 (1.63) *n = *17	− 0.21 (2.90) *n = *16	*p* = 0.835
C7 root	0.19 (1.59) *n = *16	− 1.03 (1.59) *n* = 15	*p* = 0.041
Fibular nerve at fibular head	− 0.35 (1.67) *n* = 21	− 0.03 (1.93) *n* = 17	*p* = 0.590
Fibular nerve at popliteal fossa	− 0.58 (1.32) *n* = 21	− 0.52 (1.24) *n* = 17	*p* = 0.881
Tibial nerve at tarsal tunnel	1.55 (2.44) *n* = 19	1.13 (3.25) *n* = 15	*p* = 0.670
Tibial nerve at popliteal fossa	0.92 (6.61) *n* = 20	− 1.47 (5.28) *n* = 15	*p* = 0.258
Sural nerve	− 0.02 (0.40) *n* = 21	0.08 (0.72) *n* = 17	*p* = 0.577
Sciatic nerve at mid-thigh	2.49 (7.87) *n* = 10	2.56 (6.22) *n* = 8	*p* = 0.984

*SD* standard deviation, *PN* polyneuropathy, *N* number of subjects with a CSA measurement

**Fig. 5:**
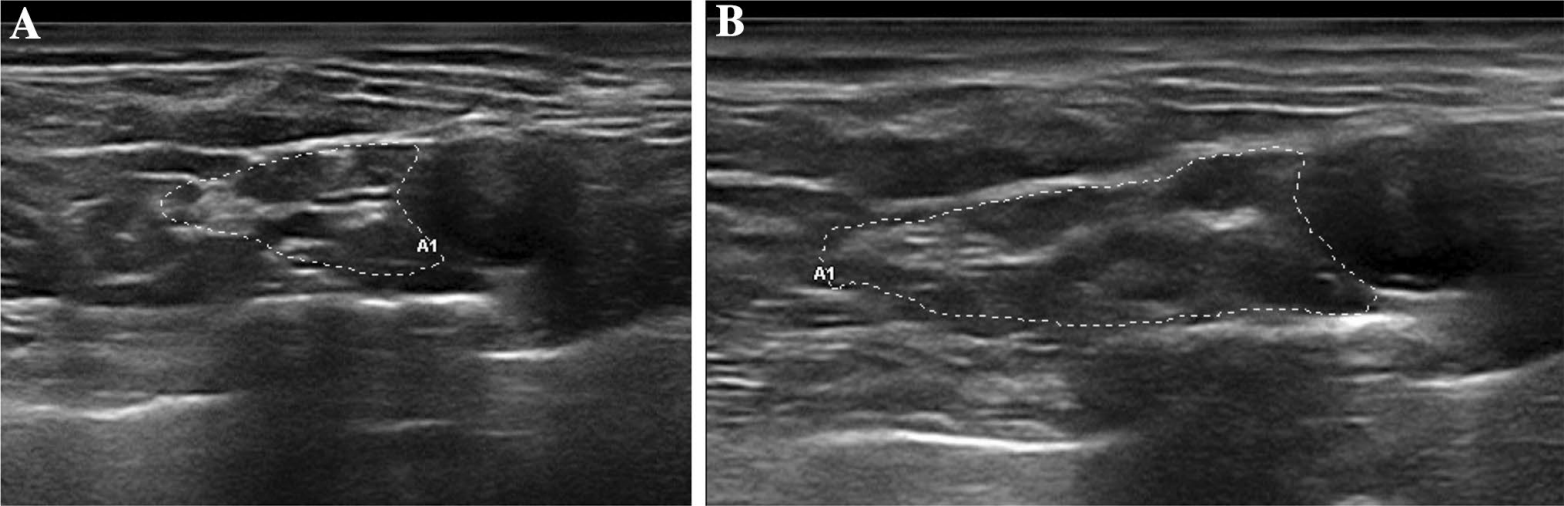
Right brachial plexus (contoured by dot lines) at supraclavicular space of a 57-year-old TTR-PN female patient. At first evaluation (**a**), brachial plexus CSA was 66 mm^2^, at second evaluation (**b**) 20 months later, CSA measured 108 mm^2^. The normal CSA value is < 82mm^2^. *ATTRv-PN* hereditary transthyretin amyloidotic polyneuropathy, *CSA* cross-sectional area

The increase of CSA at brachial plexus correlates with the length of follow-up when considering the whole cohort (*r* = 0.626, *p* = 0.005) or only the ATTRV-PN (*r* = 0.767, *p* = 0.010), while no significant correlation emerged for pre-symptomatic carriers. No significant correlation was found between NIS-LL and increase of CSA at brachial plexus (*p* = 0.318) (Fig. 5).

## Discussion

In the present study we performed, for the first time, a longitudinal nerve US evaluation in a large group of subjects (both with PN and pre-symptomatic carriers) with different TTR gene mutations. The results showed that nerve CSA progressively increases at brachial plexus. Recently, US evaluation of the same cohort at baseline revealed that nerves of ATTRv-PN patients were significantly larger than those of pre-symptomatic carriers at proximal sites (more pronounced at brachial plexus) [9]. At follow-up, nerve CSA was distinctly increased (23.8% more than the baseline value) at brachial plexus whereas at the other sites, the nerves changes (either increase or decrease) were subtle and less consistent. These findings point to brachial plexus as a hotspot of peripheral nervous system involvement in ATTRv despite the PN occurs as a length-dependent process. This unexpected distal–proximal mismatch between clinical and morphologic topography of peripheral nerve involvement is challenging. Anecdotal pathological findings demonstrated amyloid deposition in epineurium of nerve roots and in endoneurium of brachial plexus [10], therefore, it could be speculated that CSA measurement of brachial plexus at supraclavicular space may also include the connective tissue, that is more represented at this site. In addition, a previous MRI study showed how morphological nerve abnormalities cluster proximally, despite the polyneuropathy is length dependent [7]. Moreover, while the polyneuropathy is distal, the amyloid deposits are focal and preferentially located at proximal sites [10, 11]. Pathological processes caused by amyloid deposition include a space-occupying effect in the endoneurium also mediated by edema and blood vessel (*vasa nervorum*) involvement thus possibly inducing secondary ischemia of nerve fibers. It can be speculated that the co-existence of these mechanisms might explain why CSA increases proximally (amyloid deposit and edema) and polyneuropathy manifests distally (blood vessel involvement and axonal loss).

Moreover and more important, the findings of the present study show that ATTRv pre-symptomatic carriers may unveil abnormalities before clinical or neurophysiological signs occur, as already suggested from other nerve ultrastructural imaging studies [6–8]. Since brachial plexus CSA enlargement seems to represent a hallmark of ATTRv-PN, the evidence that also in pre-symptomatic carriers the CSA increases over time at the same rate as the patients with an established PN, even still within normal range, may represent a red flag of disease occurrence and progression. This finding suggests that pathological changes of peripheral nervous system initiate long before clinical evidence of PN and continues also when it becomes manifest.

Therefore, brachial plexus CSA may candidate as longitudinal biomarker of peripheral nerve involvement in ATTRv.

The present study may suffer from some limitations. The cohort reflects Italian ATTRv epidemiology, and the results require validation in endemic countries. The results are comprehensive of different mutations, and the size of each subgroup did not allow to differentiate the analyses across the different mutations. Each ATTR-PN patient had received treatment for amyloidotic PN in the course of follow-up but the type(s) and duration of treatment(s) were not considered in the present study. The duration of follow-up did not allow to observe pre-symptomatic carriers turning into ATTR-PN patients, therefore it was not possible to assess the diagnostic predictive value of the brachial plexus CSA increase rate. Moreover, the present study did not compare ATTRv-PN patients with control groups affected by other polyneuropathies, therefore, it was not possible to assess the specificity of our findings.

In conclusion, we showed that brachial plexus involvement in ATTRv patients and carriers is progressive and may be considered as a longitudinal morphological marker of disease progression both in patients, and more importantly, in pre-symptomatic carriers.

## Data Availability

Data will be shared in anonymous form on a reasoned request to the corresponding author.
